# Using multiagent modeling to forecast the spatiotemporal development of the COVID-19 pandemic in Poland

**DOI:** 10.1038/s41598-022-15605-9

**Published:** 2022-07-04

**Authors:** Piotr Pałka, Robert Olszewski, Małgorzata Kęsik-Brodacka, Agnieszka Wendland, Karolina Nowak, Urszula Szczepankowska-Bednarek, David Th. Liebers

**Affiliations:** 1 Faculty of Electronics and Information Technology, Warsaw University of Technology, Warsaw, Poland; 2 Faculty of Geodesy and Cartography, Warsaw University of Technology, Warsaw, Poland; 3 National Medicines Institute, Warsaw, Poland; 4 Department of Applied Pharmacy, Faculty of Pharmacy With Laboratory Medicine, Medical University of Warsaw, Warsaw, Poland; 5 Centre for Innovation and Technology Transfer Management, Warsaw University of Technology, Warsaw, Poland; 6 New York University Langone Health, New York, USA

**Keywords:** Environmental social sciences, Computational science, Information technology

## Abstract

In the article, the authors present a multi-agent model that simulates the development of the COVID-19 pandemic at the regional level. The developed what-if system is a multi-agent generalization of the SEIR epidemiological model, which enables predicting the pandemic's course in various regions of Poland, taking into account Poland's spatial and demographic diversity, the residents' level of mobility, and, primarily, the level of restrictions imposed and the associated compliance. The developed simulation system considers detailed topographic data and the residents' professional and private lifestyles specific to the community. A numerical agent represents each resident in the system, thus providing a highly detailed model of social interactions and the pandemic's development. The developed model, made publicly available as free software, was tested in three representative regions of Poland. As the obtained results indicate, implementing social distancing and limiting mobility is crucial for impeding a pandemic before the development of an effective vaccine. It is also essential to consider a given community's social, demographic, and topographic specificity and apply measures appropriate for a given region.

## Introduction

The novel coronavirus, severe acute respiratory syndrome coronavirus 2 (SARS-CoV-2), which causes coronavirus disease 2019 (COVID-19), has infected over 236 million people to date and contributed to more than 4.8 million deaths globally, as reported to the World Health Organization (WHO)^1^. The relatively high infectivity and long incubation period, as well as the long viral shedding period together with the current global travel pattern, constitute the key elements that allowed the SARS-CoV-2 epidemic to progress to a pandemic^2^.

Despite the implementation of unprecedented public health interventions, including social distancing and large-scale lockdowns, this infectious disease has continued to spread. However, there has been substantial variation among countries in terms of the number of waves and infections^3^. As of 8 October 2021, Poland had experienced three epidemic waves and reported approximately 3 million infection cases and nearly 76,000 deaths^1^. A fourth wave of the coronavirus epidemic is currently in progress.

The rapid spread of the epidemic and the enormous losses it has caused make understanding of the dynamics of the new pandemic disease extremely important to determine how to effectively counteract the spread. Predictive mathematical models for epidemics^4^ are crucial for understanding epidemic courses^5^, and they may be a source of information for decision makers responsible for planning effective control strategies.

Pandemic modeling can include temporal and spatial predictions^6,7^. Suspected-infected-recovered (SIR)-type classic epidemiological models mainly facilitate forecasting the number of cases over a strictly defined time interval^8^. Due to significant spatial differentiation of Poland, similar to most countries, forecasting the number of cases limited to a scalar numeric value on a given day for the entire country is wholly insufficient. Instead, as legal restrictions on can be for specific zones or geographic areas, it is crucial to model the effects of limiting economic, educational, and tourist activities and reliably model spatial epidemiological changes considering the policy instruments available.

Many methods of modeling spatial–temporal diffusion processes use approaches such as spatial interaction models, machine learning methods with multiagent modeling^9^, and multiagent modeling^10–12^. Due to relatively low spatial resolution, dividing the country into 1-km^2^ units is limited mainly to modeling at the national and regional levels^13^. It is necessary to adopt an approach that employs a different multiagent modeling method that considers interactions between the inhabitants of Polish administrative divisions to develop a reliable model of spatiotemporal changes in the spread of the COVID-19 pandemic at the local level (i.e., *powiat* or *gmina*, which is the primary unit of Poland's administrative division).

In this article, we employ agent-based modeling to forecast spatiotemporal changes in the number of COVID-19 cases, hospitalizations, and deaths at the local level in the country's *powiats*. The research aims to develop a general methodology for multi-agent modeling of the pandemic's development at the regional level, taking into account demographic and topographic factors, residents' mobility, and the adopted level of restrictions. For each administrative unit, a detailed topographic model (with the geometric accuracy level and conceptual generalization of maps at a 1:10,000 scale) was created and enriched with detailed demographic and climatic information. The authors used a set of highly differentiated criteria to select three test areas representative of different regions of Poland. The diversity of representative *powiats* allowed the generalization of conclusions for the entire country. Models of social interactions are specific to each *powiat*. The Results section contains a description of the proposed research methodology and an overview of the selection of test areas and multivariate forecast models. The Discussion section discusses the conclusions of the conducted research, while the Materials and Methods section details the developed analytical tools and the data used.

## Research methods

The authors employed an agent-based modeling (ABM) methodology to study the spread of the COVID-19 pandemic in Poland. This approach was well suited to this challenge, as it afforded the possibility of simulating complex populations in both temporal and spatial contexts while simultaneously attributing specific features to each individual in a population. ABM comprises a class of computational models that simulates the actions and interactions between autonomous individuals (called agents). These simulations usually aim to assess the impact of what is being modeled, i.e., the behavior of a single agent, on the entire system's performance, particularly on emerging outcomes.

For the implementation of ABM, the authors used the GAMA tool, which is a modeling and simulation development environment for building spatially explicit agent-based simulations^14^. GAMA has been successfully used by other researchers to simulate the spread of COVID-19^15^.

The assumption is that each agent has a set of demographic and social characteristics, spatial location, and infection status (according to the SEIAPR-DM model). During the simulation, agents that move according to the daily and weekly mobility patterns meet in a specific location. If a susceptible agent finds themself in the vicinity of an infected agent (asymptomatic or symptomatic), it is checked whether the susceptible agent is infected. The results and conclusions obtained by the authors are the outcomes of the simulations. This type of simulation is very accurate and supported by carefully selected parameters derived from the literature or relevant institutions.

The ABM methodology assumes the simulation of agents' behaviors in a specific environment, understood as a spatial reference at the level of particular *powiats*. This study used three different test areas modeled in the GIS development environment. Topographic data from an official database corresponding to a digital map with a scale of 1:10,000 were used to develop the spatial models^16^. The digital topographic model was enriched with detailed demographic data on population distribution, unemployment level, age, sex, workplaces and educational institutions, and free-time activities, among others. The model was developed based on data from the 2011 Polish census conducted by Statistics Poland^17^. Additionally, the authors carried out nonreactive research (a researcher does not interfere with the actual object of analysis). The analysis of the available data and information considered the entire range of sources, which had their origins in documenting various areas of social life and thus differed in methodological approach (qualitative research and quantitative research, including research that used GIS techniques) and spatial analysis scale (nationwide, regional and local).

The model was also enriched with meteorological data provided by the Institute of Meteorology and Water Management (IMWM)^18^; meteorological data included temperature, humidity, day length, etc., in individual powiats every day from 1 March 2020 to 31 May 2021. Statistics Poland supplied data on the mobility level of powiats' inhabitants, and Google was used for mobility analysis, broken down into six criteria. Epidemiological data collected by powiat sanitary and epidemiological stations and made available by the Information Systems Division of the Analysis and Strategy Department of the Ministry of Health^19^ was used to calibrate the model and evaluate the results.

The developed GIS model includes a comprehensive network of streets, residential and office buildings, railway lines and railway stations, factories, workplaces, shopping malls, shops, clinics and hospitals, schools, cultural institutions, places of worship, parks, and forests.

**Figure 1:**
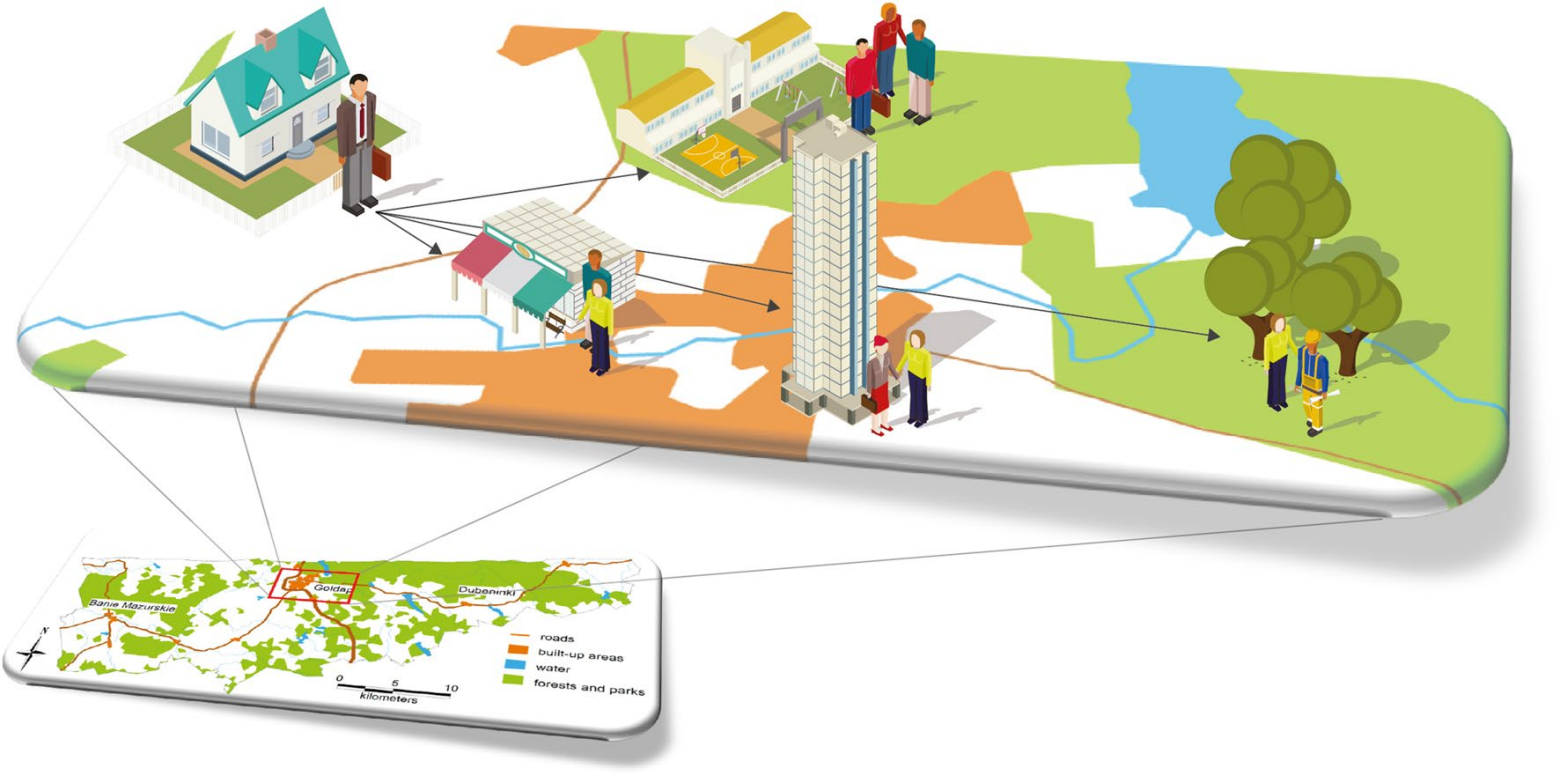
Model of an agent's spatiotemporal interactions (developed by the authors).

Each citizen living in a given *powiat* was modeled as an agent residing in a specific building, attending a specific school, working in a specific company, shopping at a local store, and receiving treatment at a specific clinic or hospital (Fig. 1).

**Figure 2:**
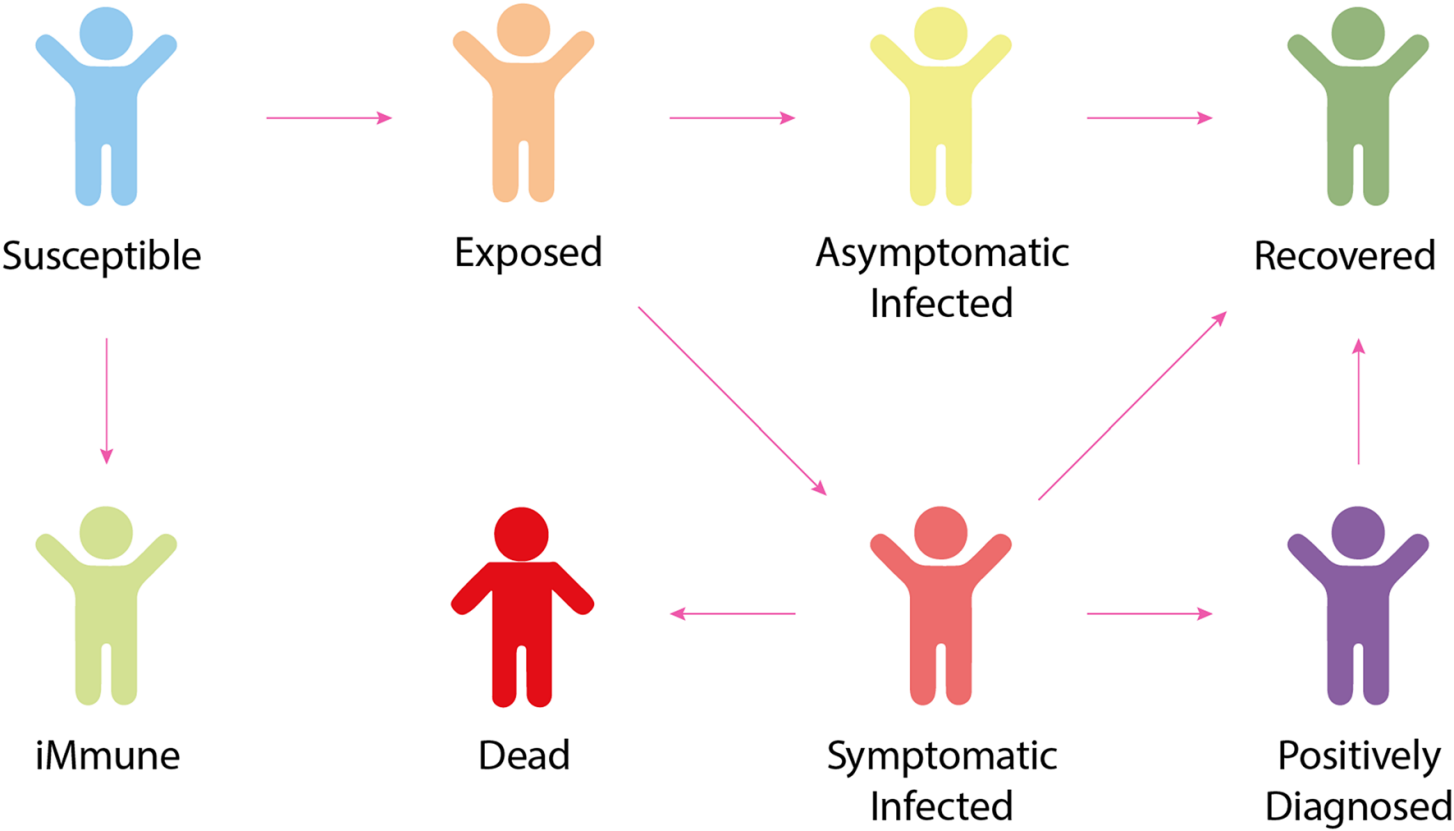
States of agent infection in the SEIAPR-DM model (developed by the authors).

The following attributes and behaviors characterize a citizen agent:

- *Demographic and social characteristics* Demographic and social characteristics include age; gender; labor market status; workplace (within the powiat of residence/outside the powiat of residence); type of residence (residential building); place of work/education (office building, factory, school); and health information, including cardiovascular disease, diabetes, obesity, etc.
- *Spatial location* An agent moves according to a lifestyle pattern of daily and weekly behaviors. On working days, the agent goes to work, school or kindergarten, stays there for a time defined individually for every person and then returns home. With decreased probability, the agent may visit cultural institutions or health centers. Over the weekend, an agent is more likely to go shopping or to a place of worship. The activity, mobility level, and traffic models are modified depending on the Stringency Index^20^. Moreover, an agent moves either on foot (distances up to 1 km) or by car/public transportation (over 1 km), depending on the distance traveled; the selected mode of transport affects the possibility of infection. The model of the agents’ movement is based on^21,22^.
- *State* The model assumes that each agent has one of the following states: immune, susceptible, exposed, infected and symptomatic, infected but asymptomatic, positively diagnosed, or dead. The transition model between these states was adopted based on previous work^23,24^ and extended to include immune status (Fig. 2). The studies^24–27^ served as the basis for selecting the parameters for: the contagiousness rate (R0) when in contact with a symptomatic or asymptomatic infected agent, the incubation period, the recovery period, time to death, and the infection mortality rate. Since the developed model is generic, it enables the consideration of many factors, such as resistance to the COVID-19 disease. The model considers an individual's immune status; the transition to this state may result from either vaccination or innate immunity. Because COVID-19 vaccination in Poland was in its infancy in the period studied by the authors, the agent's "immune" status did not play a significant role in the conducted simulations. However, thanks to the model's general nature, it is possible to perform analogous simulations for a period in which a significant (and spatially varying) percentage of the population has been vaccinated. The authors of this paper will investigate this issue in further publications.
- The *simulation* step depends on the configuration of the tool used for simulation. The experiments assumed 15-min intervals between simulations. In each step, for every susceptible agent, the model surveys whether there are infected persons in the immediate vicinity (8 m in the model), and then the probability of infection is calculated. It is contingent upon several variables: the distance between each infected agent and the target agent, whether susceptible and infected persons wear masks (and whether they do so properly), the current temperature and humidity, demographic and social characteristics (work)^28^), and whether the exposed agent was vaccinated.
- The basic model does not assume limitation of the development of a pandemic through vaccination but facilitates examination of the impact of individual topographic, demographic, and social factors and the imposed restrictions on the pace of pandemic development in particular regions.

The developed model is complex in information technology terms but relatively simple in conceptual terms. For each agent, the state variables in the model are as follows: the infection status according to the SEAIRPD-Q model, the agent's location on the map along with the agent's detailed description, the purpose of the current trip, and the mode of transport (public transport, car, walking).

The processes modeled stochastically are:

- the transitions between the states of the SEAIRPD-Q model (infection, recovery, etc.);
- the duration of the states in which the agent is found (e.g., incubation period, recovery period, time to diagnosis);
- the precise determination of the moments when the daily/weekly rhythm changes for the agent (going to work, returning home);
- the choice of, e.g., entertainment or shopping venues.

The data collected by the authors comprise the number of agents in each SEIARPD-Q state at each simulation step and the time or location of each event associated with each agent's SEIARPD-Q state change. The boundary data for each experiment comprise a detailed map of the area covered by the agents, meteorological data, stringency data, the number of agents in each state, other individual characteristics of the agent, and a set of parameters specific to each district (over a dozen). The complete model with all the data for the experiments conducted is available at: https://github.com/piotrpowerpalka/Covid-19-ABM/tree/PPbranch.

### Selection of test areas, exogenous variables, and spatial predictors

**Figure 3:**
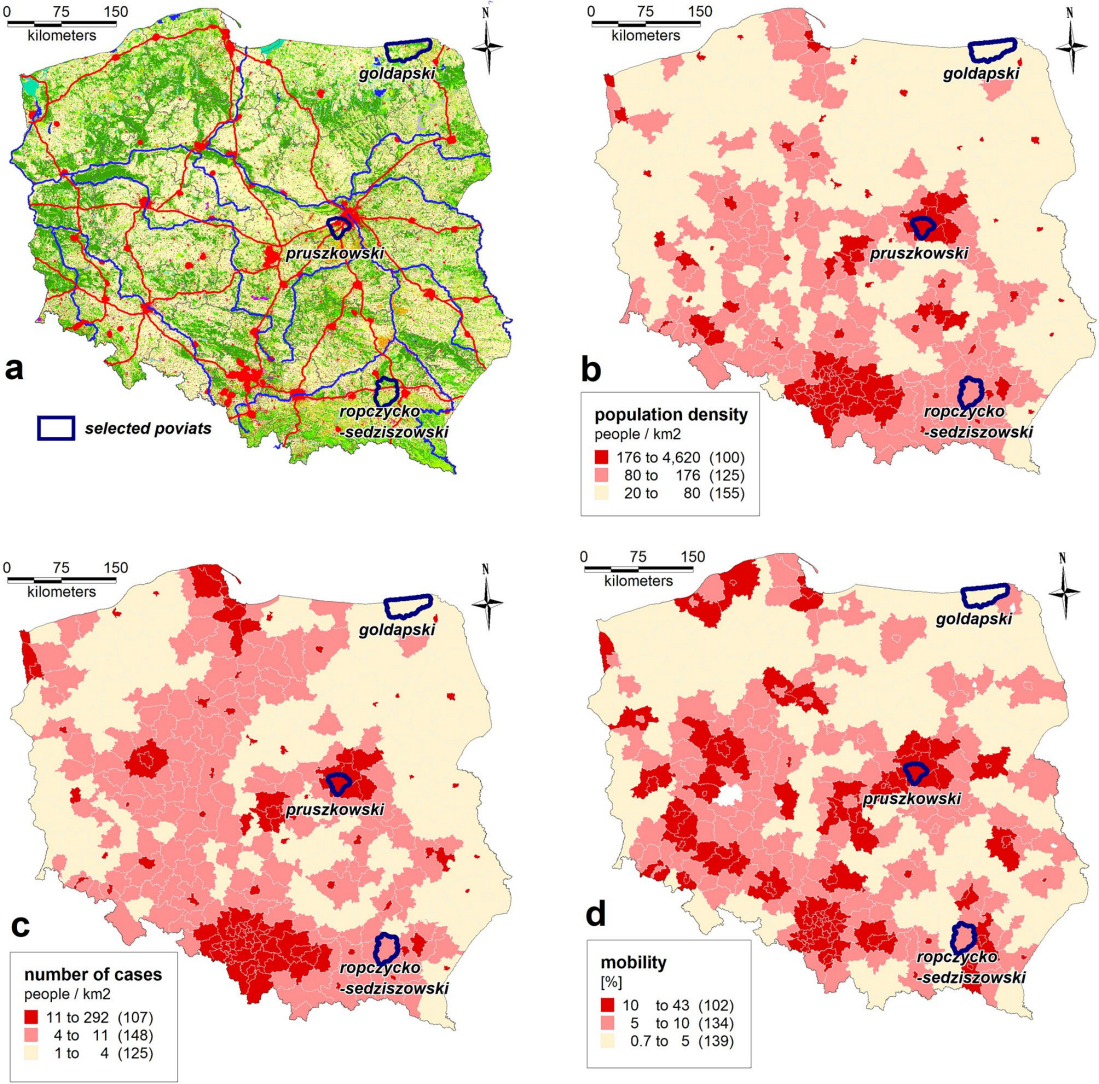
The set of selected criteria to choose the representative regions: (**a**) selected powiats, (**b**) population density, (**c**) number of cases/km^2^ and (**d**) resident mobility (developed by the authors in QGIS ver. 3.22.5).

Due to Poland's relatively significant spatial differentiation, the authors used a set of highly differentiated criteria to select three test areas representative of different regions of Poland. The factors used to characterize Poland's administrative division units (380 *powiats*) were population, population density, unemployment proportion, urban layout, transportation network density, level of public transportation use, resident mobility, climate diversity, and more extensive statistical data. Figure 3 shows the selected criteria illustrated on maps. Multicriteria analyses of exogenous variables made it possible to select the following three different *powiats*, which differ in various respects: Gołdapski (Warmian-Masurian Voivodship), Pruszkowski (Masovian Voivodeship), and Ropczycko-Sędziszowski (Subcarpathian Voivodship) (Table 1).

**Table 1:** Description of selected test areas.

*POWIAT*			
	Gołdap	Pruszków	Ropczyce-Sędziszów
Industrialization	Predominantly small and mid-size enterprises and agriculture	A large proportion of inhabitants work in the capital	A large industrial center
		Educational, cultural, and entertainment centers	
Population	Low population density (approx. 27,000 residents)	High population density (approx. 166 000 residents)	Medium population density
	Poor living conditions	Migrations to and within the Warsaw (capital) agglomeration	
	Scattered housing	High-density housing developments with high densities of residents, allowing many mutual contacts	
Transportation network and mobility of inhabitants (percentage of residents of a given *powiat* who work or study in another *powiat*)	Extremely low transportation accessibility	Spatial mobility (commuting)	East–west transportation routes traverse the *powiat*, constituting an essential element of the country's transit infrastructure
	Low mobility approx. 0.6%	Excellent connections with capital and the rest of the country	Mobility approx.6.4%
		Mobility approx. 10.1%	

The implemented model assumes that the following factors strongly correlate with disease transmission:

- the spatial distribution of the population—because the agents are spatially distributed, and the infection arises from close contact between the infected and the exposed individual;
- the residents' level of mobility—because the level of mobility may affect the number of encounters between one agent and another (potentially infected) individual;
- the characteristics of the restrictions imposed (and the compliance level)—because the model considers the extent of the restrictions, e.g., obligatory mask usage (whether an infected and exposed individual affects the likelihood of infection), compulsory remote work or education, and stay-at-home requirements affect the agent's mobility;
- weather conditions—as the probability of infection depends on the humidity and temperature of the actual day;
- the local community's specificity—because the characteristics of the local community, such as the unemployment rate, the number of people working outside their place of residence (town, commune, powiat), or religiosity, affect the community's mobility, which should result in a diversified development of infections

One should also note that due to the observable "pandemic fatigue" phenomenon, there has been a failure to comply with the restrictions and disregard of sanitary recommendations in individual test areas, prolonging the fight against COVID-19.

### Spatial data enrichment and spatial data mining

The parameters of the multiagent model used in the computational process have a dual nature, explicit or implicit, "hidden" in spatial data. A portion of the data are tabular (e.g., the dependent variable: the number of cases on a given day in a given *powiat*) or descriptive (e.g., 30% of the residents of a given *powiat* use public transportation to travel to work). However, it is necessary to transform much of the independent data used in the model from an implicit form to explicitly defined rules or parameters to operationalize its use in the model. The extraction of spatial knowledge from data was carried out during the spatial data enrichment process. For the statistical attribution of individual residents, represented by agents, to workplaces, shops, places of worship, and parks, proprietary geoinformation tools implemented in the QGIS environment have been developed. QGIS is an open-source Geographic Information System (GIS) application that supports vector, raster, and database formats, functionalities, and geospatial data analysis (qgis.org/en/site). These tools helped define the rules governing behavior in the virtual community of residents and the mutual interactions of individual agents. For instance, it was assumed that 90% of primary school students attended the school closest to their place of residence, which enabled geospatial analyses using Voronoi diagrams and digital linking of individual agents with places of residence, work, study, rest, medical care, and shopping.

Due to the considerable spatial, demographic, and economic differentiation of the selected test areas (Table 1), the process of data analysis and knowledge extraction required appropriate parameterization of the rules for each of the *powiats*.

A wide range of nonspatial data were also used to parameterize the rules characterizing individual *powiats*, including data on territorial collectivities, considering development mechanisms at the local or regional level. The authors decided on methods such as desk research and secondary statistical analysis. The authors examined human behavior, the amount of time spent working, recreating, shopping, and staying home; the professional situation; medical care; religious and consumer practices; and compliance with sanitary rules (tightened and relaxed) since March 2020.

### Parameterization of models

The authors used "The Oxford COVID-19 Government Response Tracker (OxCGRT)" to create the model. It systematically collects data on policy restrictions that have been applied in various countries to prevent COVID-19 transmission^29^. The calculations are based on the so-called Stringency Index (a composite measure based on nine response indicators, including school closures, workplace closures, and travel bans) rescaled to a value from 0 to 100 (100 = the strictest response). If policies vary at the subnational level, the index is shown as the response level of the most stringent subregion. The reasons for the analysis of the Stringency Index were its availability for most (180) countries and data availability.

The model described here is generic and can be adapted to simulation and predictive research. The primary purpose of developing the multiagent model was to enable reliable forecasting of the number of cases, hospitalizations, and deaths in particular periods in the spatially differentiated units of Poland's administrative division. This model was calibrated based on data from March 2020 to January 2021 (after this date, Poland began mass vaccinations, which significantly influenced the model). The verification of the model's operation shows that considering the characteristics of individual *powiats*, such as population distribution, mobility level, development layout, type of public transportation, and the introduced level of restrictions, facilitates a reliable forecast of the number of cases at the level of 2%.

It is possible to use this model for research purposes and verification of what-if hypotheses. In the conducted research, the authors were interested in elucidating how the spatiotemporal development of the COVID-19 pandemic in Poland would have developed if a different epidemiological restriction policy had been introduced while the demographic, topographic, and climatic conditions remained analogical. The research adopted several variants, as shown in (Table 1).

**Figure 4:**
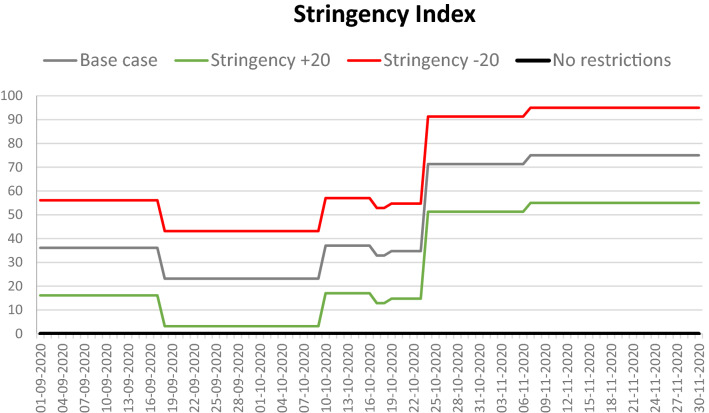
Stringency Index for Poland.

Stringency Index data^30^ served as the basis for the graph of changes in the level of restrictions enforced in Poland (Fig. 4); this served as a reference level for other models and was defined as the so-called base case (gray in charts and maps).

Stringency Index data refer to the restriction and prevention policies implemented by most countries in the world. The indicators used to calculate the index are (C1) school closings, (C2) workspace closings, (C3) public event cancelations, (C4) gathering restrictions, (C5) public transportation closings, (C6) stay at home requirements, (C7) internal movement restrictions, (C8) international travel controls, (H1) public information campaigns, (H2) testing policies, (H3) contact tracing, (H6) facial covering policies, and (H7) vaccination policies. The listed indices have integer values ranging from 0 to 2–5; their values are normalized to 1, and their mean is calculated. For more details on how the index is calculated, see^31^. The research used the following indicators for the calculations: C1–C3, C6, and H6. Applying the Stringency Index to the model changes the probability of going shopping or for a walk, going to school or work, and the number of mask wearers both inside and outside of the buildings (Table 2).

**Table 2:** The analyzed variants.

Model	Characteristics
Base case	Restrictions in line with the national policy
Stringency + 20 (PLUS)	C1–C3, C6, and H6 values are increased by 1 (if possible); this increases the level of restrictions by approximately 20%
Stringency − 20 (MINUS)	C1–C3, C6, and H6 values are decreased by 1 (if possible); this decreases the level of restrictions by approximately 20%
No restrictions	No restrictions, the situation is the same as before the epidemic: no mask mandates, no social distancing, no remote work, students attend school

## Results

The conducted research showed that adopting four variants in the models of the activity of agents in the selected test *powiats* enabled the obtainment of statistically significant results. It also enabled the precise determination of the impact of the level of restrictions on the numbers of cases, hospitalizations, and deaths. By using a geographic information system and multiagent modeling in the modeling process and a detailed database of topographic objects, it was possible to simultaneously investigate when and where an infection occurs and determine the impact of spatial location and land cover on the development of a pandemic.

To ensure the credibility of the research, the base case model was calibrated independently for all three test *powiats* based on sanitary and epidemiological data of the number of cases on individual days. The proposed model has many parameters set individually for each powiat, for instance, housing and population density, the residents' level of mobility, and the level of public transport use. Since each of these parameters may affect the accuracy of calculations in various ways, it was necessary to recalibrate the model for each powiat using source epidemiological data.

As an objective function during calibration, we assumed the minimization of the difference between the number of infected according to the real data and the model's result after 1 month of simulation. During the calibration, the assumption was that the only variable of the objective function would be the probability coefficient of infection during agent interaction. The adopted method was the bisection algorithm.

**Figure 5:**
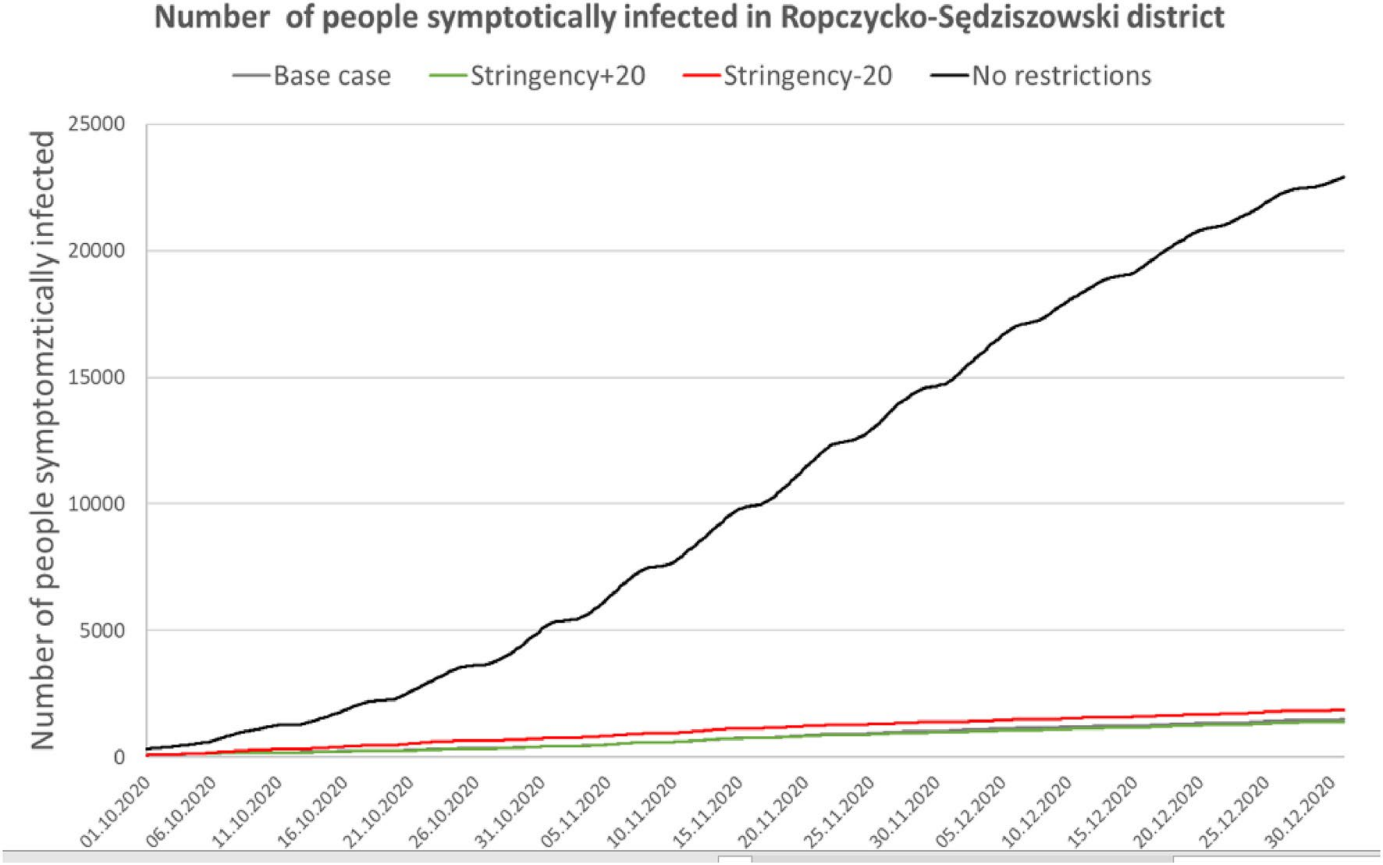
Number of symptomatic infected agents in the Ropczycko-Sędziszowski *powiat*.

**Figure 6:**
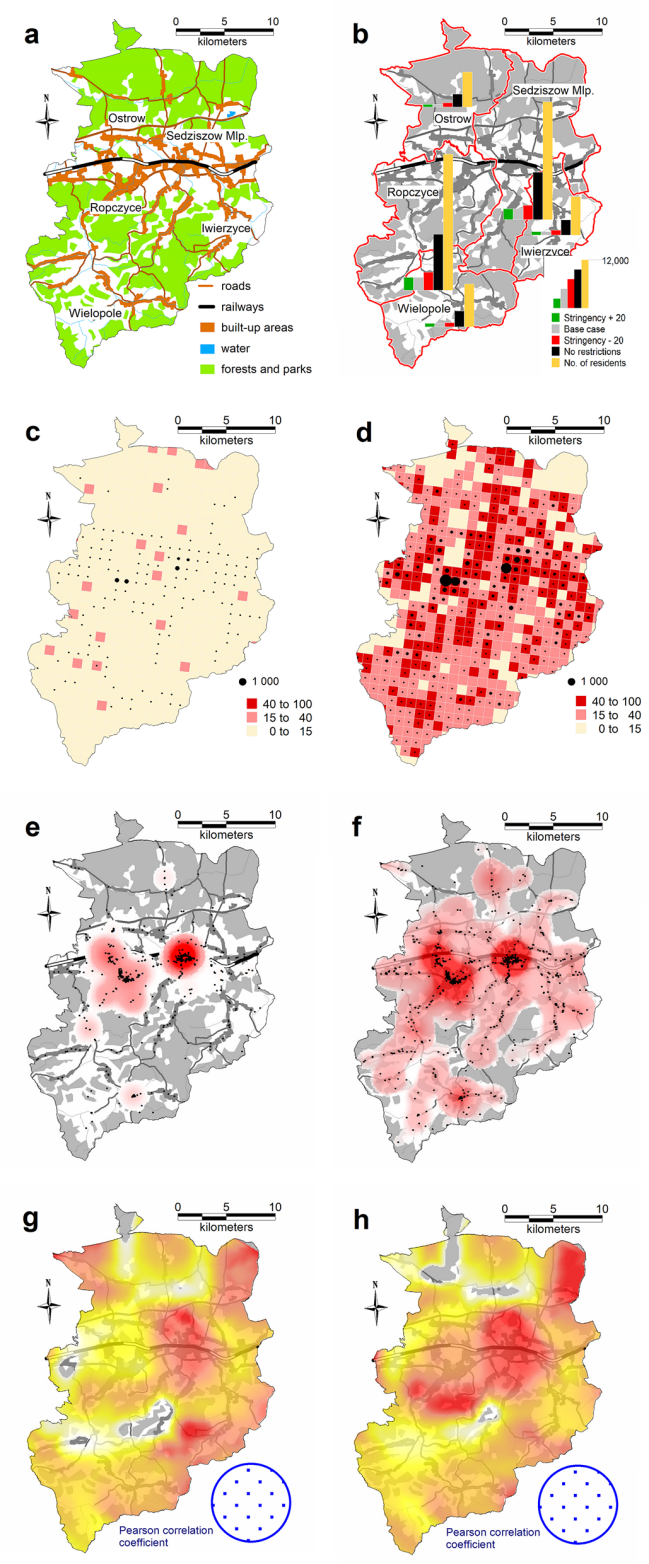
Spatial distribution of the number of cases, their locations, and the differences between individual models in the Ropczyce-Sędziszów *powiat*. (a) Ropczyce-Sędziszów *powiat*: land cover. (**b**) The number of cases in 4 models in the *gminas* in relation to the number of inhabitants of these *gminas.* (**c**) The number of cases in a 1 × 1 km grid (pie chart) and the percentage of cases (intensity of red color): the base case model. (**d**) The number of cases in a 1 × 1 km grid (pie chart) and the percentage of cases (intensity of red color): the no restrictions model. (**e**) The location of cases (black dots) and the density of cases by place of infection: the base case model. (**f**) The location of cases (black dots) and the density of cases by place of infection: the no restrictions model. (**g**) The Pearson correlation coefficient between the place of infection and the number of inhabitants in the area (white < 0.5, yellow < 0.75, red ≥ 0.75): the base case model. (**h**) The Pearson correlation coefficient between the place of infection and the number of inhabitants in the area (white < 0.5, yellow < 0.75, red ≥ 0.75): the no restrictions model (developed by the authors in QGIS ver. 3.22.5).

**Figure 7:**
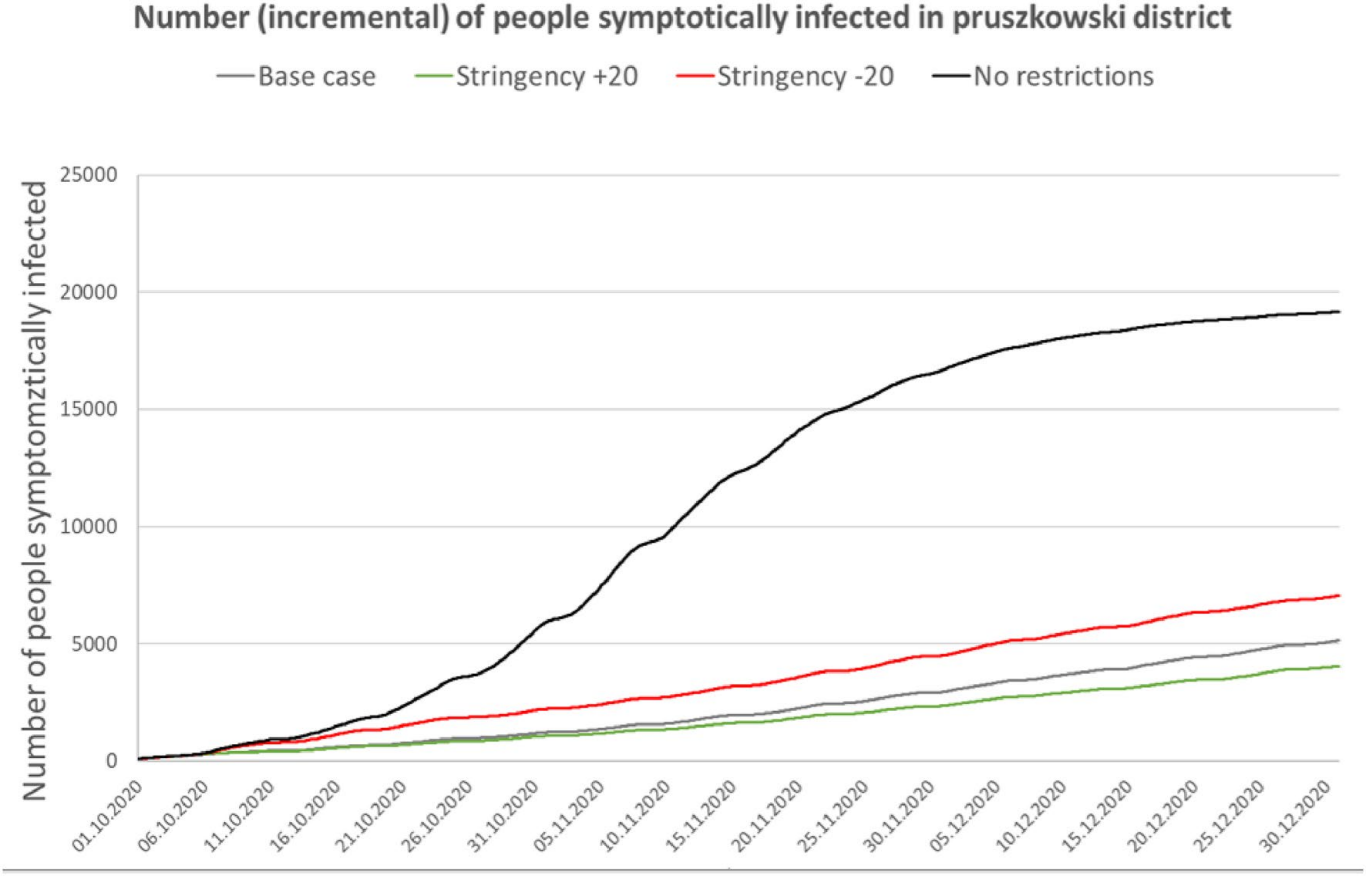
Number of symptomatic infected agents in the Pruszków *powiat.*

**Figure 8:**
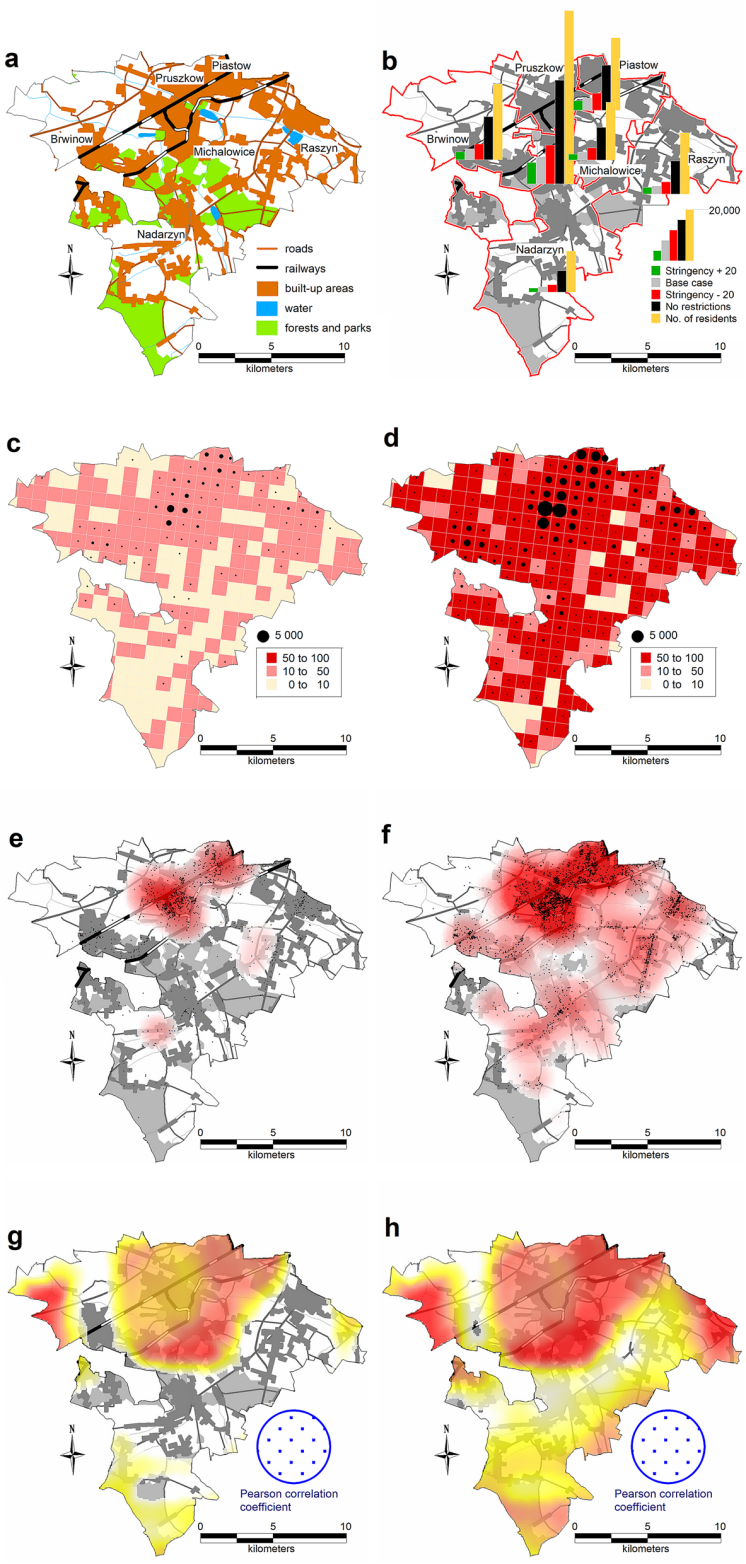
Spatial distributions of the number of cases, their locations, and differences between the individual models in the Pruszków powiat. (**a**) Pruszków powiat: land cover. (**b**) The number of cases in the 4 models in the *gminas* in relation to the number of inhabitants of these *gminas. (****c****)* The number of cases in a 1 × 1 km grid (pie chart) and the percentage of cases (intensity of red color): *Stringency Index + 20* model. (**d**) The number of cases in a 1 × 1 km grid (pie chart) and the percentage of cases (intensity of red color): *No restrictions* model. (**e**) The location of cases (black dots) and the density of cases by place of infection: *Stringency Index + 20* model. (**f**) The location of cases (black dots) and the density of cases by place of infection: *No restrictions* model. (**g**) The Pearson correlation coefficient between the place of infection (developed by the authors in QGIS ver. 3.22.5).

**Figure 9:**
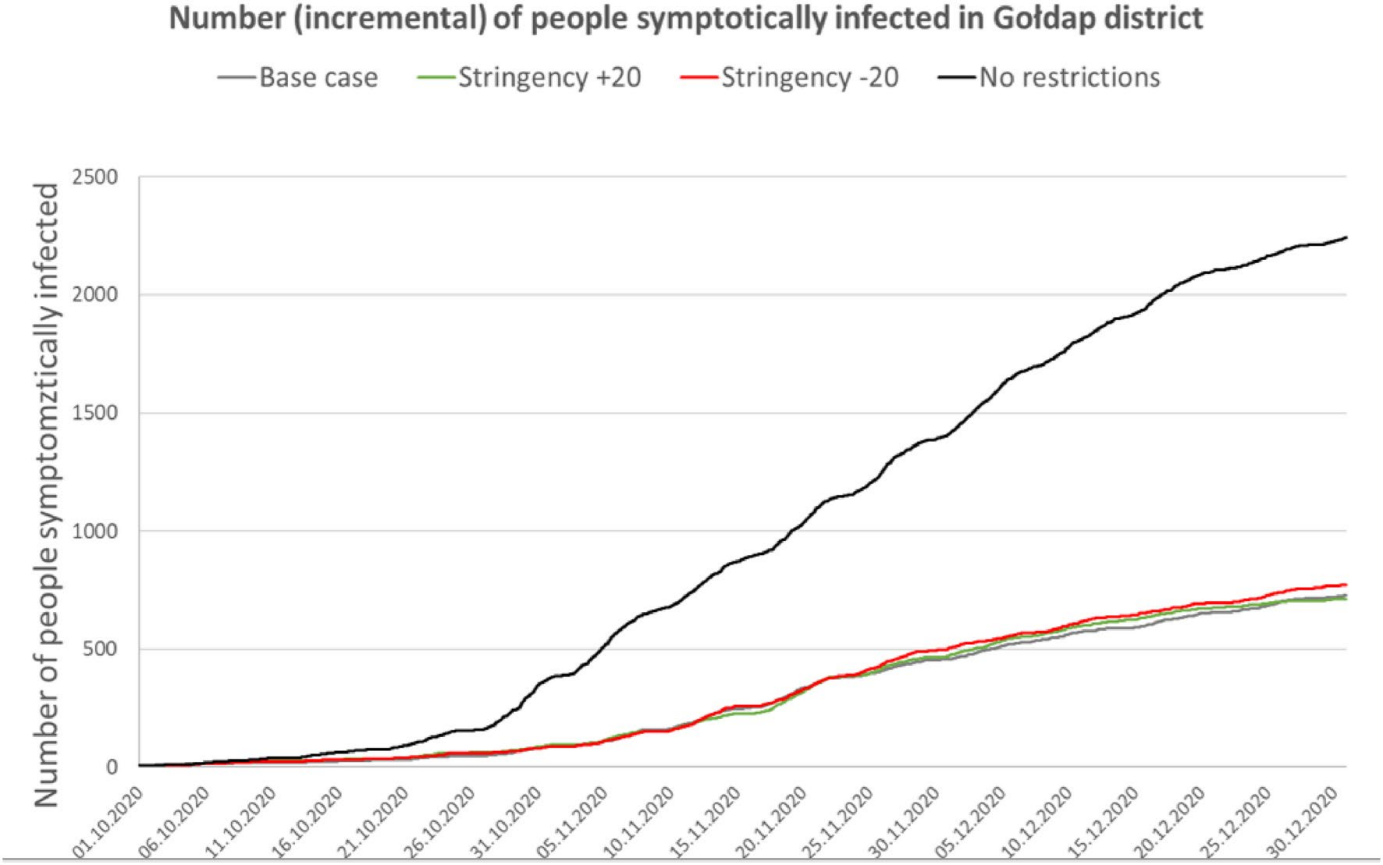
Number of symptomatic infected agents in the Gołdap *powiat.*

**Figure 10:**
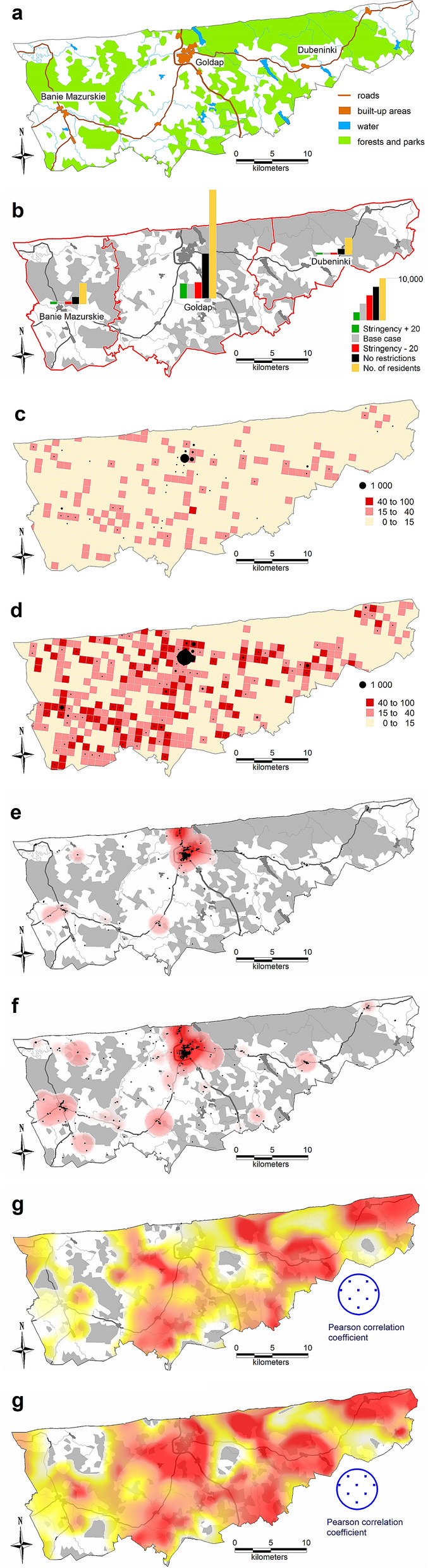
Spatial distributions of the numbers of cases, their locations, and the differences between the individual models in the Gołdap powiat. (**a**) Gołdap powiat: land cover. (**b**) The number of cases in the 4 models in the *gminas* in relation to the number of inhabitants of these *gminas. *(**c**) The number of cases in a 1 × 1 km grid (pie chart) and the percentage of cases (intensity of red color): *Stringency Index − 20* model. (**d**) The number of cases in a 1 × 1 km grid (pie chart) and the percentage of cases (intensity of red color): *No restrictions* model. (**e**) The location of cases (black dots) and the density of cases by the place of infection: *Stringency Index − 20* model. (**f**) The location of cases (black dots) and the density of cases by place of infection: *No restrictions* model. (**g**) The Pearson correlation coefficient between place of infection and the number of inhabitants of the area (white < 0.5, yellow < 0.75, red ≥ 0.75): *Stringency Index − 20* model. (**h**) The Pearson correlation coefficient between the place of infection and the number of inhabitants of the area (white < 0.5, yellow < 0.75, red ≥ 0.75): *No restrictions* model (developed by the authors in QGIS ver. 3.22.5).

For the Gołdap *powiat*, the accuracy (MPE error) was 1.64%; for Ropczyce-Sędziszów, the accuracy was 0.68%; and for Pruszków, the accuracy was 0.64%. Due to the model’s high computational complexity, the calibration process was nontrivial; thus, the researchers did not choose complete automation of the process. The probability coefficient of infection during agent contact was calibrated. Because of the nature of the model, this coefficient required only minor adjustments to calibrate to actual data. However, depending on the *powiat*, the calibration process took several days to 2 weeks. Tables 3, 4 and Figs. 5, 6, 7, 8 and 9 present the numerical results obtained by the multiagent model, while Figs. 6, 8 and 10 show the spatial distribution of the number of cases, their locations, and differences between individual models.

**Table 3:** Numerical results obtained in the multiagent model for the tested powiats.

	Base	Plus 20	Minus 20	No rules
	No. of cases	No. of cases	No. of cases	No. of cases
**Gołdap *powiat***				
Asymptomatic infected	2912	2960	3173	8747
Symptomatic infected	722	706	767	2237
Exposed	3677	3690	3982	11,182
Recovered	3272	3373	3542	9991
Positively diagnosed	359	355	398	1119
Positively diagnosed and hospitalized	21	29	31	82
Symptomatic infected and hospitalized	21	15	20	44
Dead	7	3	11	12
Total	10,991	11,131	11,924	33,414
**Pruszków *powiat***				
Asymptomatic infected	19,642	15,708	27,876	76,264
Symptomatic infected	5058	3952	6968	19,054
Exposed	24,895	19,567	35,175	94,769
Recovered	21,810	17,478	31,343	93,779
Positively diagnosed	2427	1966	3459	9577
Positively diagnosed and hospitalized	210	141	280	777
Symptomatic infected and hospitalized	104	74	119	357
Dead	36	26	72	171
Total	74,182	58,912	105,292	294,748
**Ropczyce-Sędziszów *powiat***				
Asymptomatic infected	5406	5391	7226	22,602
Symptomatic infected	1407	1324	1782	5599
Exposed	6720	6561	8979	28,639
Recovered	6338	6326	8334	25,525
Positively diagnosed	708	663	910	2747
Positively diagnosed and hospitalized	68	54	71	201
Symptomatic infected and hospitalized	36	25	48	102
Dead	9	9	16	50
Total	20,692	20,353	27,366	85,465

**Table 4:** Numerical results obtained in the multiagent model for the selected powiats: place of infection.

		Base		Plus 20		Change	Minus 20		Change	No rules		Change
		No. of cases	[%]	No. of cases	[%]	[%]	No. of cases	[%]	[%]	No. of cases	%	[%]
**Gołdap *powiat***												
Infection in/at	Buildings	264	7.2	310	8.4	17.4	262	6.6	− 0.8	1283	11.5	386.0
	Commercial facilities	698	19.0	667	18.1	− 4.4	837	21.0	19.9	3598	32.2	415.5
	Work	2696	73.3	2710	73.4	0.5	2871	72.1	6.5	6166	55.1	128.7
	Schools	0	0.0	0	0.0		0	0.0		43	0.4	
	Public transportation	12	0.3	0	0.0		0	0.0		38	0.3	
	Recreation	7	0.2	3	0.1		12	0.3		54	0.5	
	Total	3677		3690		0.4	3982		8.3	11,182		204.0
**Pruszków *powiat***												
Infection in/at	Buildings	1918	7.7	1803	9.2	− 6.0	2164	6.2	12.8	7364	7.8	283.9
	Commercial facilities	2082	8.4	679	3.5	− 67.4	5330	15.2	156.0	22,287	23.5	970.5
	Work	16,231	65.2	12,488	63.8	− 23.1	20,627	58.6	27.1	50,251	53.0	209.6
	Schools	91	0.4	25	0.1	− 72.5	102	0.3	12.1	635	0.7	597.8
	Public transportation	52	0.2	13	0.1		61	0.2		212	0.2	
	Recreation	435	1.7	629	3.2	44.6	548	1.6	26.0	5738	6.1	1219.1
	Transport	4086	16.4	3930	20.1		6343	18.0		8282	8.7	
	Total	24,895		19,567		− 21.4	35,175		41.3	94,769		280.7
**Ropczyce-Sędziszów *powiat***												
Infection in/at	Buildings	205	3.1	248	3.8	21.0	289	3.2	41.0	1196	4.2	483.4
	Commercial facilities	894	13.3	748	11.4	− 16.3	1243	13.8	39.0	5875	20.5	557.2
	Work	4408	65.6	4345	66.2	− 1.4	5207	58.0	18.1	17,347	60.6	293.5
	Schools	7	0.1	3	0.0	− 57.1	12	0.1	71.4	47	0.2	571.4
	Public transport	8	0.1	5	0.1		12	0.1		34	0.1	
	Recreation	35	0.5	21	0.3	− 40.0	52	0.6	48.6	593	2.1	1594.3
	Transportation	1163	17.3	1191	18.2	0.2	2164	24.1	86.1	3547	12.4	304.9
	Total	6720		6561		− 2.4	8979		33.6	28,639		326.0

It is worth noting that the process of multiagent modeling, which included tens of thousands of agents interacting with each other in a virtual topographic space with a level of detail (LoD) corresponding to analog maps at a 1:10,000 scale, was lengthy and computationally demanding. The calculations were performed in the CENAGIS computing cluster with 16 Intel (R) Xeon (R) Silver 4216 CPU @ 2.10 GHz processors with 128 GB RAM. The calculations for a single case for the base variant took 5 h 25 m for the Gołdap *powiat*, 10 h 34 m for the Ropczyce-Sędziszów *powiat*, and 33 h 45 m for the Pruszków *powiat*. It should be stressed that the spatial interpretation of the obtained results required complex SQL querying of the database with spatial operators. To highlight various aspects of the obtained results for individual models, the authors provide a summary for entire *powiat*s, *gminas* constituting *powiats*, and 1 km^2^ units, as used in the official statistics. Additionally, to analyze the spatial relationship between individual parameters, e.g., the number of people living in a given region and the number of cases in this area, the authors used proprietary tools to determine the Pearson correlation coefficient in a moving (circular) window of a given size. The authors obtained discrete results (e.g., point information on the percentage of incidence in a 1 km^2^ area) and interpolated them to show a continuous statistical surface illustrating the spatial distribution of individual phenomena (Figs. 6, 8 and 10).

When analyzing the data in Table 3, it should be emphasized that due to the specificity of the multiagent model used, the analysis of "exposed" cases is of crucial importance. "Exposed" means contact with the potential to cause infection; some infected agents will develop symptoms characteristic of infection only after the incubation period (median incubation period of 5.1 days^27^). This is important because not only the moment when the symptoms arise but also the moment of infection are considered, and the duration between such events may be several to several dozen days. Naturally, the numbers of symptomatic infected, asymptomatic infected, hospitalized, and deceased agents are also essential for analysis. The results (Table above) indicate that changing the level of restrictions significantly affects the number of cases. Obviously, due to the different number of inhabitants in individual *powiats*, it is crucial to compare the values between particular models. In almost all cases, the incidence rate in the PLUS model is several percentage points lower than that in the base case. The opposite was observed for the MINUS model with a lower level of restrictions. The no restrictions model showed very significant differences, sometimes exceeding the reference value by threefold. The results show the importance of restrictions such as social distancing, remote learning, movement limitations, and mask use.

It is interesting to compare the spatial distribution of the "exposed" locations. While the analysis indicates the role of the level of restrictions, it also reveals the impact of topography, building density, recreational areas, quality of public transportation, and resident mobility. The three analyzed *powiats* represent individual regions of Poland and enable the determination of the impact of topographic factors on the course of the pandemic. As the table below (belowTable 4) shows, the inhabitants of the Gołdap *powiat* have a level of mobility close to zero, and there is almost no public transportation. As a result, the number of cases related to traveling does not exceed 0.3%. In the Pruszków *powiat*, where a approximately 12% of inhabitants commute to the capital every day (mainly by suburban railway), the level of infection connected to public transportation reaches 20.1% in the PLUS model. In the MINUS model for the Ropczyce-Sędziszów *powiat*, which has good connections to the city of Rzeszów, the percentage of cases related to public transportation exceeds 24%.

Workplaces account for the highest percentages in the number of cases:

- 73.4% in the Gołdap *powiat* (the PLUS model),
- 65.2% for the base case in the Pruszków *powiat*,
- 66.2% in the Ropczyce-Sędziszów *powiat* (the PLUS model).

It should be emphasized that depending on the model, the number of people who become infected at their workplace differ significantly. Mobility restrictions and requisite remote work may increase the number of infections related to recreational areas (city parks and forests). In the Pruszków *powiat*, characterized by a relatively small area of parks, applying more significant restrictions (the PLUS model) causes a percentage decrease in the number of cases in particular places from 6% (buildings) to 75% (clinics), except for recreational facilities, which are associated with an increase in the number of cases (compared to the base case) by 45% (!). What is equally significant is the percentage increase in the number of cases related to commercial facilities in this *powiat* in the MINUS model (156% of the base case model) and the no restrictions model (970% of the reference value), demonstrating the vital role of shops and malls in spreading COVID-19 when there are no social distancing restrictions in place.

The conducted analysis also shows that infection occurs in residential buildings in nearly 10% of cases. Introducing restrictions leads to almost complete elimination of cases related to public health care facilities (0.1% in the PLUS model in the Pruszków *powiat*), with very low absolute values (5 people in the Ropczyce-Sędziszów *powiat* in the PLUS model and 13 people in the Pruszków *powiat* in the same analytical variant). Closing schools and transitioning to remote learning are also of great importance; lack of such restrictions resulted in 43 infected students in the Gołdap *powiat*, 47 in the Ropczyce-Sędziszów *powiat*, and 635 in the Pruszków *powiat*, with 0, 7, and 91 corresponding values in the base model and 0, 3, and 25 in the PLUS model, respectively.

The maps show the results of the no restrictions model and one of the models including the spatial distancing policy (Figs. 6, 8 and 10), illustrating the differences resulting from topographic or demographic differentiation and from adopting a specific restrictive policy for each of the three analyzed *powiats*. For the Ropczyce-Sędziszów *powiat*, the maps show the base case. For the Pruszków *powiat*, the maps show the Stringency Index + 20 (the PLUS variant). For the Gołdap *powiat*, the maps show the Stringency Index − 20 (the MINUS variant). Such an approach enables the analysis of the spatial differentiation of the development of the COVID-19 pandemic and the verification of research hypotheses indicating the crucial role of restrictive policies.

### Ropczyce-Sędziszów *powiat*

The no restrictions model for this *powiat* had the highest increase in the total number of infections (326% compared to the base model) out of all the analyzed *powiats*. The greatest increase in the number of infections occurred in recreational areas (as high as 1594%). The bar chart in Fig. 6b shows the absolute values of the “exposed” agents in the individual models in relation to the number of inhabitants of individual *gminas* comprising the *powiat*. The values related to the no restrictions model are dominant; the bar sizes indicate two towns, Ropczyce and Sędziszów Małopolski, with the highest number of cases.

The maps in Fig. 6c,d show a different approach: the number of cases in individual 1 km^2^ units (the size of the pie chart) and the percentage of people who fell ill in a given areal unit in relation to the number of inhabitants of a given square. One should note that this analysis shows the number of infected people living in a given square unit, regardless of the place of infection. Comparison of the base case and the no restrictions models shows considerable differentiation in the disease prevalence and incidence. In the absence of restrictions, over 40% of the inhabitants who fell ill occupied approximately 1/3 of the powiat's area. In the reference model (base case), the value exceeds 15% for only a dozen areal units. Black dots on the maps in Fig. 6e,f show where infections occurred, while the statistical surface layer indicates the number of cases in a given region, represented by varying intensities of red. This map also shows the primary role of areas with dense industrial or residential development in the progression of the pandemic. The maps (Fig. 6g,h) show a linear Pearson correlation between where infections occurred and the number of inhabitants in that area. The correlation coefficient value is calculated in a moving window with a radius of 2.5 km, which serves as a spatial filter. In a given areal unit, the values of the number of cases and the number of inhabitants in the individual squares of the official statistical grid are analyzed. The obtained point values (discrete) are then interpolated to a continuous statistical surface. White indicates no correlation, yellow indicates a weak linear correlation (Pearson's correlation coefficient of 0.5), and red indicates a strong correlation (correlation coefficient > 0.75). The strongest spatial correlations occur in densely populated areas where many people fall ill. When analyzing the obtained results, the level of spatial generalization of the results should be considered; each dot on the map represents a value assigned to its circular surroundings with a radius of 2500 m (nearly 20 km^2^).

The map (Fig. 6) reveals interesting conclusions: in both models, infection cases in Sędziszów Małopolski are more concentrated, while infection cases in Ropczyce are more dispersed. One way to explain this is that Sędziszów Małopolski is smaller and less populated than Ropczyce, but the population density is double (Sędziszów Małopolski: 838 people/km^2^, Ropczyce: 336 people/km^2^), with a railway station in its center.

### Pruszków *powiat*

The Pruszków *powiat* is inhabited by the largest number of people, with high labor mobility. A significant number of the inhabitants commute to work in neighboring areas (mainly Warsaw) via public transportation (Warsaw Commuter Railway, train); therefore, public transportation and work constitute the most significant infection sources. Additionally, in this *powiat*, the highest percentage increase in infections in all the analyzed models occurred further from the house (more than 3 km); accordingly, the proportion of people who become infected at home is lower than those in the other analyzed *powiats*. In the no restrictions model, the highest increase in infections was recorded in recreational areas (1219% of the base case model value), while in the MINUS model, the highest increase in infections was recorded in trade-related areas. On the other hand, in the PLUS model, despite a significant decrease in the number of infections compared to the base variant (by an average of 21.4%), there was an increase in the number of infections in recreational areas (45%). The reason is the increase in professional restrictions (remote work and learning) and the population's willingness to visit open natural areas.

As seen in the maps in Fig. 8, in the case of Pruszków *powiat*, the authors present the results of the no restrictions model and the most restrictive PLUS model. The pie charts show that the highest number of cases occur in the main urban centers, Pruszków and Piastów, and more than half of the Pruszków *powiat* contains areas in which over 50% of residents will fall ill in the no restrictions model. Moreover, in the no restrictions model, regions with high infection densities are strongly correlated with places with high population densities (Brwinów, Raszyn, Nadarzyn, Michałowice). Notably, the largest numbers of railway stations are in the *gminas* of Pruszków, Piastów, and Brwinów. Interestingly, there were many infections in the former two *gminas*, while the latter *gmina* (Brwinów) had the smallest number of infections. Brwinów has a low degree of industrialization, and the existing industrial centers (mainly warehouses) are located outside the city.

Comparison of the maps in Fig. 8 indicates that the model in which the level of safety was increased by 20% in relation to the restrictions implemented in Poland (the PLUS variant) showed a significant reduction in the number of cases and complete elimination in areas with an incidence rate higher than 50%. The no restrictions model has a stronger correlation between the number of cases in a given area and the population density, which is evident in the eastern (Michałowice and Raszyn), western (Brwinów), and southern (Nadarzyn) parts of the *powiat*. In the PLUS model, the correlation is almost zero, while it exceeds 0.5 in the no restrictions model.

### Gołdap *powiat*

The Gołdap *powiat* has the smallest population and the lowest level of resident mobility; most residents work on their own farms. Consequently, this *powiat* had the lowest total number of infections in all the analyzed scenarios. In relation to the base model, there is an increase in the number of infections in the no restrictions model by 204% (the smallest increase among all the analyzed *powiats*). Contrary to the other *gminas*, there is no significant increase in the number of infections in recreational areas (0.5%). The MINUS model shows the smallest increase in infections (8.3% in total) compared to the base model. On the other hand, only in the Gołdap *powiat* is the PLUS model characterized by a slight increase (0.4% on average) in infections (in the other *powiats*, the total number of infections in this model decrease).

The conducted analyses show that among the three *gminas* that make up the Gołdap powiat, a significant increase in the number of cases occurs mainly in the town of Gołdap (Fig. 10b). It should be emphasized that the comparison of the modeling results in Fig. 10 relates to the analysis of two models with low levels of restrictions: the Stringency Index -20 (MINUS) and the no restrictions models. In both models, the primary infection outbreaks occur in the *powiat*'s capital, where infections occur at home, at work, in shops and in schools. Due to the agricultural nature of this *powiat*, characterized by scattered housing developments and low resident mobility, the overall number of cases is relatively low, even in the model without restrictions. However, the percentage of infections in some units of the 1 km^2^ statistical grid exceeds 40%, indicating significant roles of topography, scattered single-family housing, the level of economic development of the region, and the mobility of residents over the course of the pandemic.

## Discussion, conclusions, and future work

The developed research methodology makes it possible to model many states of the agent, i.e., the various phases of the agent's illness, while taking into account the spatial location of individuals, the type or intensity of their interactions, and the conditions stemming from the degree of restrictions in place and the spatial environment. Thanks to its structure, the system analyzes the impact and the interplay between individual factors on the pandemic's development. The developed tool, a multi-agent modeling system, enables analyzing any area for which demographic and topographic data, information on the residents' mobility, and the level of imposed epidemiological restrictions are known. Thus, the proposed approach facilitates performing simulations on a national scale or forecasting the number of cases in areas with different population density, mobility, and public transport usage. This analysis, performed for different variants, may prove extremely useful for regional and national authorities ahead of the subsequent season of disease incidence.

The article's authors presented their results to the Polish Ministry of Health several times. The results have played a part in modifying the approach to counteracting the development of subsequent phases of the pandemic by utilizing spatial and demographic differentiation of the country and taking measures appropriate to the local conditions.

This research has shown that the adoption and consistent application of restrictions such as mask mandates, spatial distancing, remote working and learning, and resident mobility limitations are all crucial in modeling the progression of the COVID-19 pandemic. In the model with no restrictions, the number of cases is 2 to 3 times larger than that in the base model. This difference is even larger with a level of restriction lower than that at baseline. Other studies have shown that cloth face coverings alone can significantly reduce person-to-person virus transmission and reduce the daily growth rate of COVID-19 infections by 40–60%^32^.

Our data indicate that people’s interactions in workplaces have the largest predicted impact on virus spread. As many as 50–70% of infections, depending on the variant and the powiat, are associated with the workplace. Commercial facilities, which are usually crowded, are also places in which there is a high probability of virus transmission. Our results showed that in the base model, 8.4% of individuals were infected while shopping indoors; with no restrictions, this percentage increased by 23.5%, and with restrictions, it increased by 20%, and the number of infected agents dropped to only 3.5%.

Our results suggest that virus transmission is also influenced by age, unemployment percentage and resident mobility. We found that virus transmission among people staying at home is twice as low (4.2%) and among people working outside the powiat is almost three times as high (26.7%) as those in the base model, with an overall infection rate of 9.4%. In the MINUS model, these disproportions are even greater, at 7.2%, 48.7% and 12.5%, respectively. Other research has provided consistent evidence suggesting that mobility is correlated with the transmission intensity of SARS-CoV-2 over time in several countries^33^. These results support the implementation of social distancing interventions to control the epidemic.

Ensuring reliable results requires effective modeling methods and reliable source data that allow system calibration. The use of multiagent modeling systems enables complete parameterization of the modeling process. The parameterization considers agents' behaviors under specified sanitary regime conditions and the full use of digital topographic data, with a high LoD. The current study has shown that the impact of topographic conditions, density of housing developments, type and size of workplace facilities, number and size of commercial facilities, organization of public transportation, and size and availability of recreational areas all significantly impact the course of the pandemic. A more urban population is predicted to have a lager number of cases. In general, urban environments are recognized as risk factors for the transmission of respiratory pathogens^34^. Other studies have also found an association between urban areas, high-density populations and the number of COVID-19 cases as well as earlier detection of COVID-19^3^. Hence, measures to reduce viral transmission, focusing on social distancing in particular, are reasonable in the control of COVID-19 transmission^35^.

The obtained results show that the adopted level of restrictions is the most crucial factor in the pandemic's development. Therefore, hindering the pandemic requires limiting the residents' mobility, minimizing the number of interactions, and strictly adhering to mask usage and disinfection recommendations. Applying these practices may reduce the number of cases in particular regions severalfold. Also, as it is crucial to ensure society's physical and mental well-being during a pandemic, the authors deem it advisable to broaden the use of open spaces, such as forest areas, for the residents' recreational use.

The conducted research has also shown that:

- The use of reliable topographic, demographic, social, and sanitary and epidemiological data makes it possible to calibrate the base case model at a level of error equal to 1%, enabling reliable what-if analyses that utilize alternative models.
- The developed calculation model is a complex simulation tool that takes into account several parameters and determinant factors for each person (age, gender, place of residence or work), space (place of residence or work, shops, parks, clinics, transportation networks, etc.), conditions (weather data, restriction policies), resulting in a complex epidemiological model. Supplying the model with relevant data for a specific location (city, *gmina*, *powiat*, voivodeship, country) enables the simulation of the progression of the epidemic. The performed calculations concerned three representative Polish *powiats* (Gołdap: 27,595, Ropczyce-Sędziszów: 71,697, and Pruszków: 162,547 inhabitants) and required a strong machine to perform the calculations; a more substantial computing cluster would enable detailed calculations for larger areas, e.g., voivodships or the entire country.
- The Stringency Index is a helpful tool that parameterizes restriction policies, standardizing the model for any region, country, and time. However, one should emphasize that this index is based on only a few indicators, and its scaling is nonlinear. The no restrictions model, which allowed free movement of residents and did not consider any restrictions, produced a significantly larger number of cases than any of the models with at least minimal restrictions in terms of distancing, mask use, etc.
- The GAMA platform used in the research proved to be an efficient and convenient modeling environment for ABM, which uses digital spatial data with a high LoD. What comfirms its efficiency is its ability to carry out calculations with a time resolution of 15 m, lasting 3 months, for a *powiat* with 162,547 inhabitants, represented by moving and interacting agents. It should also be stressed that obtaining the final results requires synergy between GAMA and GIS tools, enabling the postprocessing of spatial queries to the database and visualizing results in spatially localized maps and diagrams.

To summarize the conducted research, the results are promising and prove the model's usefulness for what-if analyses. Due to the use of topographic, demographic, sanitary and epidemiological data, the reliability of the obtained results is satisfactory. Using data for three spatially different *powiats* that are simultaneously representative of different regions of Poland enables a straightforward generalization of the model for the whole country. Understanding how combinations of factors affect the course of a pandemic will help in predicting the spread of COVID-19 and determining the right intervention directions.

The obtained results reveal the critical role of the adopted level of restrictions in limiting the pandemic's development. However, it has also been fascinating to analyze other factors influencing the course of the pandemic in various regions. For instance, the developed model demonstrated the significance of using public space and public transport (particularly apparent in the case of the commuter rail in the Pruszków powiat). Using forest areas and parks for the recreation of residents significantly reduces the number of cases by minimizing the number of interactions in a large area. It is a significant result because local authorities banned from using forest areas in the initial period of the COVID-19 pandemic in Poland. It is also essential to study other factors influencing the seasonal course of a pandemic, such as changing atmospheric conditions.

The need to adopt the most effective interventions to combat infection is now critical due to declining population immunity and new SARS-CoV-2 variants of concern (VOCs), which have higher infectivity, greater severity and antigenic escape abilities^36^.

In the present stage of the pandemic, the governments of many countries, including Poland, transitioned from a national lockdown to localized interventions^37^. This was due to the heavy socioeconomic cost of total lockdown, which was introduced at the start of the pandemic. The model we have developed, based on the use of ABM, can be a highly useful tool for determining the probability of infection transmission in a given region and an information resource for authorities who need to introduce specific restrictions to prevent virus transmission.

Moreover, the model and its four model variations are flexible, and it is possible to adapt them to emerging SARS-CoV-2 variants. Due to the specificity of the transmission of respiratory pathogens in general, our model can be used to predict not only the progression of the SARS-CoV-2 pandemic but also the transmission of other infectious diseases caused by respiratory viruses.

Further work will consider the application of various vaccination policies, which will answer the question concerning the optimal spatial policy in terms of slowing down the progression of the pandemic and its effective control.

## Data Availability

The datasets generated and analysed during the current study are available in the repository: https://github.com/piotrpowerpalka/Covid-19-ABM/tree/PPbranch.
